# An efficient annotation and gene-expression derivation tool for Illumina Solexa datasets

**DOI:** 10.1186/1756-0500-3-183

**Published:** 2010-07-02

**Authors:** Parsa Hosseini, Arianne Tremblay, Benjamin F Matthews, Nadim W Alkharouf

**Affiliations:** 1 United States Department of Agriculture, Soybean Genomics and Improvement Laboratory, 10300 Baltimore Ave, Beltsville, MD, 20705, USA; 2 Jess and Mildred Fisher College of Science and Mathematics, Department of Computer and Information Sciences, Towson University, 7800 York Road, Towson, Maryland, 21252, USA

## Abstract

**Background:**

The data produced by an Illumina flow cell with all eight lanes occupied, produces well over a terabyte worth of images with gigabytes of reads following sequence alignment. The ability to translate such reads into meaningful annotation is therefore of great concern and importance. Very easily, one can get flooded with such a great volume of textual, unannotated data irrespective of read quality or size. CASAVA, a optional analysis tool for Illumina sequencing experiments, enables the ability to understand INDEL detection, SNP information, and allele calling. To not only extract from such analysis, a measure of gene expression in the form of tag-counts, but furthermore to annotate such reads is therefore of significant value.

**Findings:**

We developed TASE (Tag counting and Analysis of Solexa Experiments), a rapid tag-counting and annotation software tool specifically designed for Illumina CASAVA sequencing datasets. Developed in Java and deployed using jTDS JDBC driver and a SQL Server backend, TASE provides an extremely fast means of calculating gene expression through tag-counts while annotating sequenced reads with the gene's presumed function, from any given CASAVA-build. Such a build is generated for both DNA and RNA sequencing. Analysis is broken into two distinct components: DNA sequence or read concatenation, followed by tag-counting and annotation. The end result produces output containing the homology-based functional annotation and respective gene expression measure signifying how many times sequenced reads were found within the genomic ranges of functional annotations.

**Conclusions:**

TASE is a powerful tool to facilitate the process of annotating a given Illumina Solexa sequencing dataset. Our results indicate that both homology-based annotation and tag-count analysis are achieved in very efficient times, providing researchers to delve deep in a given CASAVA-build and maximize information extraction from a sequencing dataset. TASE is specially designed to translate sequence data in a CASAVA-build into functional annotations while producing corresponding gene expression measurements. Achieving such analysis is executed in an ultrafast and highly efficient manner, whether the analysis be a single-read or paired-end sequencing experiment. TASE is a user-friendly and freely available application, allowing rapid analysis and annotation of any given Illumina Solexa sequencing dataset with ease.

## Background

In one run, the Illumina Solexa Genome Analyzer II sequencer produces over 50 billion nucleotides of DNA sequence data [1]. The Illumina Solexa sequencer can be used to sequence genomes as well as sequence DNA reverse transcribed from RNA to provide gene expression information. As the read length of Illumina Solexa sequencing increases, mainly due to advancements in its chemistry, so too does the volume of data generated from sequencing experiments. What may have taken months to sequence many years ago now takes days, with the additional bonus of unprecedented genome depth. However with such rapid turnaround-time comes its own set of challenges. First, terabytes of storage space is required for the resultant data, and in order to analyze such datasets, high powered computing infrastructure is required to extract and make sense of the data [2,3]. Furthermore, analysis of lesser popular sequenced organisms such as plants, including fruits, and vegetables, is not supported by Illumina's GenomeStudio [4], proving to make post-sequencing analysis even more challenging.

With Solexa sequencing, the output from the sequencer is initially in the form of .tiff (Tagged Image File Format) images [2]. These images go through a pipeline known as the GenomeAnalyzer (Illumina, Inc), developed specifically for performing three major functions: image analysis, base-calling and genome alignment. Alternatives to the GenomeAnalyzer however do exist, such as Swift [5]. By the end of the GenomeAnalyzer pipeline, the GenomeAnalyzer would have performed alignments with the sequenced reads and a reference genome with accompanying DNA sequence quality scores [2]. Furthermore, third-party tools exist which map sequenced reads onto a reference genome [6,7]. An optional fourth component, CASAVA, takes the newly generated GenomeAnalyzer alignments and performs SNP detection, allele calling and INDEL detection, amongst many other features [2]. From this analysis, a CASAVA-build is produced, containing the sequenced DNA reads which are separated into folders representing the specific chromosome they are located in. The CASAVA-build is compatible with Illumina's GenomeStudio software package were the CASAVA-build can be visualized with greater depth while gaining deeper insight into features such as understanding INDELs, SNP information, exon splice variants and junctions. However the genomes of many organisms do not have the necessary prerequisite files to be in a format compatible with GenomeStudio. Such compatibly is determined by whether necessary organism-specific prerequisite files are available on the USCS Genome Browser [8].

The CASAVA-build organizes and stores reads in directories which represent the chromosomes of the sequenced organism [1]. The directories are further divided into 10 mega base increments such that the reads found within that 10 mega base genomic range are placed in that particular sub-folder [2]. Manually organizing DNA reads within the build is error prone since every chromosome is represented with a directory, and within that are additional sub-folders to represent DNA reads broken-up into 10 mega base windows. Human error can be eliminated by developing an automated method to store all the reads into a given file of which represents all the reads in the chromosome. Therefore, knowing that each chromosome is represented by a directory, a viable approach to eliminating user-error is by traversing the sub-folders of the chromosome's directory and concatenating all the sequenced DNA reads into a single file. This file contains all the reads found in the chromosomes directory, except it eliminates the need for having numerous sub-folders and additional files. Using publicly available genome and functional annotations, sequenced reads are iteratively annotated. Following suit, a measure of gene expression known as tag-counting is employed which calculates the number of synthesized DNA sequenced being found between functionally annotated regions. Herein, we propose TASE, or Tag counting and Analysis of Solexa Experiments, a database-driven Java GUI, which accomplishes this by performing read concatenation, tag-counting and the analysis of Illumina datasets in an ultrafast and highly efficient manner, especially useful for organisms with genomes not supported by Illumina GenomeStudio.

## Methods

### Implementation

TASE is written in Java and the Java Swing user-interface library. We chose Java and Swing due to its ease and robust nature for developing user-interface applications. TASE uses Microsoft SQL Server database management system [9], serving as a data-store for both the chromosomes in the given lane and the annotation files for the given sequenced organism. TASE interfaces with SQL Server using the jTDS JDBC driver [10]; a fast Java database driver utilized to enable the calculation of tag-count and derivation of functional annotations. TASE also graphically represents chromosomal reads per lane using the JFreeChart graphing library [11].

### Concatenation of reads

**Figure 1:**
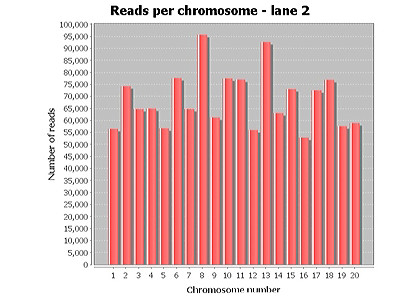
**Reads per chromosome**. The number of sequenced reads per CASAVA folder is concatenated, calculated and visually presented before annotation is performed. Dataset from Tremblay, A. (2010).

TASE analysis is divided into two distinct but yet highly related phases: DNA read concatenation for each given chromosome per selected lane of interest, followed by gene expression calculations using tag count measurements and homology-based annotation. To initiate analysis, a successfully generated CASAVA-build must first be present. Within this build, the 'export' directory contains folders for all the chromosomes pertaining to the sequenced organism, and its contents are what drive the analysis [2]. Upon defining a CASAVA-build, the contents of the 'export' folder are recursively traversed, iterating through all the sub-folders which represent chromosomes. In doing so, all the DNA reads for the given chromosome are appended to its own respective file. Therefore the number of reads for all the sub-folder will equal that in the respective chromosome file. The index of the read of which signifies its locations within a given chromosome is also appended alongside the DNA sequence; proving crucial in the eventual stage of deriving functional annotations and calculating tag-counts. Other properties such as the Illumina Solexa hardware ID, direction of the sequence (forward or reverse), and flow cell lane number, are also saved to the file. Bar graphs are produced for all lanes selected for analysis which illustrate the number of DNA reads per chromosomes (Figure 1).

### Measuring gene expression using tag-counts, and functional annotation

A set of two tab-delimited text files are required to initiate tag-count analysis and functional annotations, respectively:

1) Genomic start and end sites: Must contain genomic start and end sites for genes pertaining to the sequenced organism. The base-pair ranges will be used to perform tag-count analysis.

2) Homology-based annotations: There must also be annotations corresponding to the genomic start and end sites. The annotations are used in assigning gene functional annotations based on homology.

**Figure 2:**
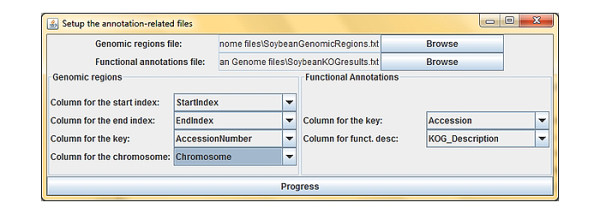
**Defining the necessary columns for tag-counting and analysis**. There must be a column in both files which have like values. Above, such a column is 'Accession' and 'AccessionNumber'.

Both files serve a critical role in analysis: Gene expression relies on counting the number of DNA sequences that fall within the range of the start and end sites of a gene, i.e. tag-counting. TASE takes the two user-defined files and performs table-querying between them, producing a joined-table containing the start and the end of the translated portion of the gene (ORF), as well as the respective functional annotation pertaining to that given genomic range. Therefore there must be attributes common between the two files to enable successful table-joining to occur (Figure 2), or else both tag-count analysis and gene annotations will produce inaccurate output. Such annotation files are readily available for many organisms in public repositories such as organism-specific databases pertaining to the sequenced organism. For example, both files representing the functional annotations and defined gene-encoding regions for *Glycine max *(Soybean) were found on the DOE JGI *Glycine max *ftp [12]. An experimental dataset for use in TASE was obtained by Tremblay et. al [13].

Distinct column names must be present in the first line of both files, due to the fact that they are important aspects of both tag-counting and annotation derivation. Upon selection of the two files, a dialog is presented in which it is divided in two halves: each for the two required files. A total of six selections are to be made which conclude which columns from the first line in the two files are to represent the start site and end site index, the keys (to be used between the two files), the chromosome and finally, the column containing functional annotations (Figure 2).

After the necessary columns are selected, a dialog is presented to enable a connection to an SQL Server instance, prompting for the server username and password. The dialog prompts also for the server instance as well as the class-driver for the SQL Server JDBC driver. By default, the class-driver for jTDS is automatically inserted. After successful login, a database is created using Java and jTDS, named after the TASE project name entered upon first starting TASE. Following suit and utilizing the jTDS JDBC driver, both the gene-encoding ranges and functional annotations files are bulk-imported to the newly created database. All the files representing the chromosomes, concatenated earlier, are also bulk-imported into the same database. Each file, whether it represents a chromosome or one of the user-defined files, have their contents stored in their own physical table. Depending on the processor speed and system specifications, database bulk-upload time will vary.

Once upload to the database is complete, a dialog appears which contains all the chromosome files which were uploaded. Clicking any chromosome name within this list will initialize both tag-count calculation and functional annotation derivation. Upon such a click, SQL code is automatically generated which interacts with the jTDS driver and SQL Server to ultimately execute the analysis for a given chromosome. The following algorithm serves as the basis behind both tag-count analysis and functional-annotation derivation:

for each chromosome selected for analysis:

*extract and store its DNA read indices*.

*tag count = number of times RNA-Seq reads are found in-between all annotations start and end site*.

*retrieve the homology-based annotation for the corresponding tag-count, based on the columns specified as shared between the two files*.

continue

write output to file

**Figure 3:**
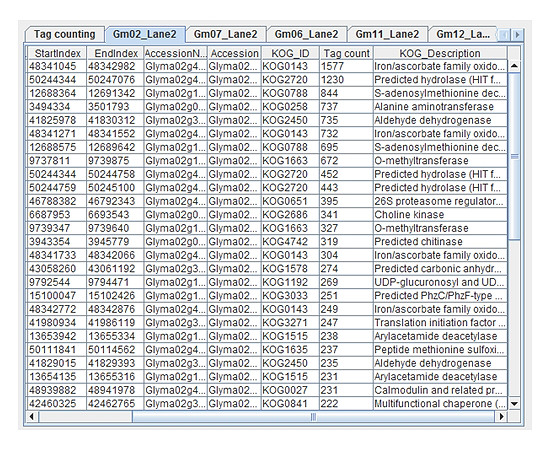
**Generated output**. Resultant output is displayed visually and saved locally.

For any selected chromosome, the resultant output is saved as a tab-delimited text file with the following notation: {chromosome}_{lane #}.txt. The files are saved in the 'output' folder of the TASE project directory created while running TASE. Generated output is also displayed in tabs, enabling an opportunity to view the top 50 annotations sorted by tag-count (Figure 3). Furthermore, the output file contains all the columns in both the functional annotations and gene-encoding region files, with the addition of tag-count measurements to signify gene-expression values per annotation.

## Findings

TASE has high computational efficiency, both in-terms of analysis time and tag-counting. To measure performance, we utilized soybean (*Glycine max*) data in which all eight lanes of the Illumina flow cell were utilized [13] (Table 1).

**Table 1:** Number of reads per lane

Chromosome	Chrom size (bp)	Lane1	Lane2	Lane3	Lane4	Lane5	Lane6	Lane7	Lane8
1	55,915,595	44087	56460	144310	125574	CONTROL	73721	77785	90255

2	51,656,713	58000	74229	190042	165634	CONTROL	96910	102557	117524

3	47,781,076	50677	64715	164496	142812	CONTROL	84010	88910	101738

4	49,243,852	50726	64908	165724	144360	CONTROL	84645	89600	102509

5	41,936,504	44080	56686	144930	125480	CONTROL	73587	78373	89551

6	50,722,821	61200	77619	199609	173280	CONTROL	100638	106572	122915

7	44,683,157	50451	64728	165386	144206	CONTROL	84790	89232	102316

8	46,995,532	75392	95643	245844	214476	CONTROL	125271	133156	152202

9	46,843,750	48079	61227	157808	138132	CONTROL	80633	84986	97566

10	50,969,635	61068	77456	198859	172297	CONTROL	101868	107309	122479

11	39,172,790	60306	77010	195521	170430	CONTROL	100450	105876	120835

12	40,113,140	43131	55977	141515	123466	CONTROL	72493	76784	88282

13	44,408,971	72442	92603	235446	204937	CONTROL	119710	126250	146480

14	49,711,204	49088	63027	161172	140375	CONTROL	82670	86971	99934

15	50,939,160	57040	73027	186523	162741	CONTROL	95461	100196	114936

16	37,397,385	41074	52851	134554	117510	CONTROL	68635	72143	82947

17	41,906,774	56466	72499	186013	161964	CONTROL	94513	99838	113529

18	62,308,140	59460	76866	195838	170495	CONTROL	100206	105912	121360

19	50,589,441	45240	57579	146309	127167	CONTROL	74770	79226	90339

20	46,773,167	46146	58890	150392	131338	CONTROL	76533	80716	92677

***Reads aligned to genome***		*1,074,153*	*1,374,000*	*3,510,291*	*3,056,674*	-	*1,791,514*	*1,892,392*	*2,170,374*

***Reads with annotations***		*640,467*	*851,595*	*2,297,371*	*1,970,284*	-	*1,101,214*	*1,150,125*	*1,363,662*

***Reads without annotation***		*433,686*	*522,405*	*1,212,920*	*1,086,390*	-	*690,300*	*742,267*	*806,712*

Lanes 1 and 2 had one pM of cDNA, lanes 3 and 4 had 4 pM of cDNA while lanes 6, 7 and 8 had 2 pM of cDNA. The number of reads is roughly proportional to the cDNA concentration. The number of reads per lane which aligned to the Soybean genome is provided. The number of reads which had functional annotation is also provided. Figure 1 ratifies the textual data pertaining to lane 2 in this table.

TASE was executed using the soybean genome build 1.0 [14]. A Python script was developed to extract the DNA sequence out of files representing individual chromosomes. Functional annotations and gene locations were retrieved from the DOE JGI *Glycine max *website [12]. The Soybean genome is approximately 1115 mega bases [15,16] and 7 of the 8 flow cell lanes had well over one million reads [13]. Lane 5 is an Illumina control [13]. For the other lanes, all reads contained Asian Soybean Rust (ASR); skewing the actual number of Soybean-only DNA reads [13]. Regardless, TASE was more than capable of analyzing all eight lanes with ease; handling the analysis of a single lane in no more than 7 minutes (Table 2). All tests were run on a dual-core 2 gigabyte CPU personal notebook with 4 gigabytes RAM, Windows 7 OS and SQL Server 2008 Developer Edition.

**Table 2:** Performance testing TASE

#/lanes analyzed	Specific lane(s)	#/chromosomes w/reads	Total #/reads	Read concatenation (min:sec)	Tag counting, annotation (min:sec)
1	2	20	1,374,000	0:59	3:17

1	4	20	3,056,674	2:02	4:56

4	1,3,7,8	80	8,647,210	8:04	11:52

8	Entire flow cell	160	14,869,398	12:13	15:55

Numerous tests were performed to measure the efficiency of TASE using datasets of varying sizes.

Performance was tested using data from 4 of the 8 lanes of the flow cell. All lanes had well over 1 million reads, with a read length of 39 base-pairs (Table 2). However, regardless of the sheer number of reads, TASE performed read concatenation, annotation and tag-counting results in less than 20 minutes (Table 2). However analysis time is proportional to genome size. Therefore, analysis times will vary for organisms with larger or smaller genomes.

The analysis time for one lane was no more than 7 minutes (Table 2). As additional lanes are added to the workload, time necessary to not only concatenate but also perform tag-counting and annotation increases in a linear fashion.

In a traditional Illumina sequencing experiment, there is usually one lane dedicated as a control [2]. Due to there being minimal DNA reads, TASE analyzes this lane in a matter of seconds, cutting the tag-counting and annotation time possibly by several minutes or even more.

## Conclusions

We developed TASE (Tag counting and Analysis of Solexa Experiments), a rapid tag-counting and annotation GUI-based software tool specifically designed for Illumina sequencing datasets. Developed in Java and deployed using jTDS JDBC driver and a SQL Server backend, TASE provides an extremely fast means of calculating gene expression through tag-counts while annotating sequenced reads with the gene's presumed function, from any given CASAVA-build. Though TASE is developed for Windows operating systems with SQL Server, however its packaged jTDS JDBC driver provides compatibility with Sybase database management systems in non-Windows operating systems. Such a build is generated for both DNA and RNA sequencing. Analysis is broken into two distinct components: DNA sequence or read concatenation, followed by tag-counting and annotation. The end result produces output containing the functional annotation and respective gene expression measure signifying how many times sequenced reads were found within the genomic ranges of functional annotations. TASE is a powerful GUI tool, free of a command-line prompt, with the intent to facilitate the process of annotating a given Illumina Solexa sequencing dataset. Our results indicate that both functional annotation and tag-count analysis are achieved in very efficient times, providing researchers to delve deep in a given CASAVA-build and maximize information extraction from a sequencing dataset.

## Availability

Project name: TASE (Tag counting and Analysis of Solexa Experiments)

Project homepage: http://sourceforge.net/projects/tase/

Operating Systems: Windows

Programming languages: Java SE 1.6, Java Swing

Other requirements: Microsoft SQL Server 6.5, 7, 2000, 2005, 2008

License: GNU General Public License v3 (GPLv3)

## Abbreviations

CASAVA: Consensus Assessment of Sequence and Variation.

## Competing interests

The authors have no competing interests. This work was funded in part by the United Soybean Board under grant 7258. Mention of trade names or commercial products in this article is solely for the purpose of providing specific information and does not imply recommendation or endorsement by the United States Department of Agriculture.

## Authors' contributions

PH developed the TASE application, graphical user interface and application logic. NA and BM supervised work, reviewed manuscript, application and algorithm. AT tested TASE and provided advice on the algorithm. All authors read and approve the final manuscript.
